# Notch3 inhibits epithelial–mesenchymal transition by activating Kibra-mediated Hippo/YAP signaling in breast cancer epithelial cells

**DOI:** 10.1038/oncsis.2016.67

**Published:** 2016-11-14

**Authors:** X Zhang, X Liu, J Luo, W Xiao, X Ye, M Chen, Y Li, G-J Zhang

**Affiliations:** 1 Changjiang Scholar's Laboratory, Cancer Hospital of Shantou University Medical College, Shantou, China; 2 The Breast Center, Cancer Hospital of Shantou University Medical College, Shantou, China

## Abstract

Invasion, metastasis and chemoresistance are leading causes of death in breast cancer patients. A vital change of epithelial cells, epithelial–mesenchymal transition (EMT), is involved in these processes. Unfortunately, the molecular mechanisms controlling EMT remain to be elucidated. Our previous studies have shown that ectopic N3ICD expression inhibits EMT in MDA-MB-231, a triple-negative breast cancer (TNBC) epithelial cell line. To decipher the mechanism, we performed in-depth studies. Specifically, we found that overexpressing N3ICD transcriptionally upregulated the expression of Kibra, an upstream member of the Hippo pathway. Correspondingly, we also observed that phosphorylated Hippo pathway core kinases, including Lats1/2 and MST1/2, were increased and decreased by overexpressing and knocking down Notch3, respectively. Furthermore, we found that the oncogenic transcriptional coactivator yes-associated protein (YAP), which is negatively regulated by the Hippo pathway, was inhibited by overexpressing N3ICD in breast cancer epithelial cells. The ability of Kibra to inhibit EMT has been previously reported. We thus speculated that Notch3 inhibition of EMT is mediated by upregulated Kibra. To verify this hypothesis, a rescue experiment was performed. Evidently, the ability of Notch3 to inhibit EMT can be countered by knocking down Kibra expression. These data suggest that Notch3 inhibits EMT by activating the Hippo/YAP pathway by upregulating Kibra in breast cancer epithelial cells, and Kibra may be a downstream effector of Notch3. These findings deepen our understanding of EMT in both development and disease, and will undoubtedly help to provide new therapeutic strategies for interfering with cancer invasion and metastasis, especially for TNBC.

## Introduction

Breast cancer affects the lives of millions, and has become a major health problem in China and worldwide. Although many scientific advancements and a great deal of progress have been made in breast cancer research such that the chances of disease-free survival for breast cancer survivors has increased tremendously over the last few decades, most patients with breast cancer cannot escape eventual recurrence, metastasis and chemoresistance, because breast cancer is a heterogeneous disease characterized by different molecular drivers. Therefore, outcomes are drastically different for each individual cancer, particularly for triple-negative breast cancer (TNBC) patients with an aggressive clinical course, early relapse and decreased survival.

It remains extremely challenging to cope with recurrence, metastasis and chemoresistance. The epithelial–mesenchymal transition (EMT) is a critical biological process during embryonic development that endows epithelial malignant tumor with the increased abilities of motility and invasiveness, chemoresistance and radioresistance.^1^ It is therefore considered the probable first key step in the complex processes of chemoresistance, local recurrence and distant metastasis.^2, 3, 4^ Over the last few decades, the mechanisms of EMT initiation and progression have been widely studied, and a number of hypotheses have been proposed^5, 6^ including multiple oncogenic events, important signaling pathways, cancer stem cells and miRNA. For example, transforming growth factor-β (TGF-β)/Wnt/Notch/hepatocyte growth factor signaling,^7, 8, 9, 10^ oncogenic Src or Ras activation,^1^ cancer stem cells,^11, 12, 13^ miRNA^14^ and inflammation^15^ are all implicated in the induction of EMT, but the exact molecular mechanism of EMT and the key genes that drive EMT remain unknown. Thus, a comprehensive understanding of the molecular mechanisms and discovering ‘driver genes' for breast cancer recurrence and metastasis are vital for recently proposed precision medicine.

Notch is a well-known, evolutionarily conserved signaling pathway that has an important role in a variety of biological processes including stem cell maintenance, differentiation, proliferation, motility, survival and cell fate specification during development. Emerging evidence indicates that Notch signaling has a critical role in mammary development,^16^ mammary stem cell function and luminal fate commitment.^17, 18^ The Notch signaling pathway is considered an important regulator of EMT induction.^19^ Furthermore, Notch activity has been suggested to correlate with proliferation, anti-apoptotic signaling and tumor progression in breast cancer.^20^

One recent study has shown that each Notch family member may target different downstream genes. Notch paralogs may even have contrasting roles in the same tissue. Notch1 and Notch2 have opposite effects on embryonal brain tumor growth through activation of different target genes.^21^ Notch1 may act as an oncogene^22^ and Notch2 may have a tumor-suppressor role in different stages of human breast cancer.^23^

A study from our group showed that Notch3, but not Notch1, can upregulate ERα expression levels (unpublished data), and, furthermore, ERα can inhibit EMT by suppressing Bmi1 in breast cancer cell lines.^24^ These results indirectly suggested that Notch3 can inhibit EMT in breast cancer cells. Unfortunately, the molecular mechanism by which Notch3 inhibits EMT has not been successfully deciphered. Exploring key molecules and mechanisms is necessary, and may be used to design new targeted drugs for managing breast cancer recurrence and metastasis.

Here, we present solid evidence that Notch3 can act as a tumor suppressor in breast cancer epithelial cells, that the loss of Notch3 is an important feature of TNBC, and that Notch3 inhibits EMT by activating the Hippo/yes-associated protein (YAP) pathway mediated by Kibra in breast cancer.

## Results

### Tumor cell lines with high relative malignancy present lower Notch3 expression levels

**Figure 1:**
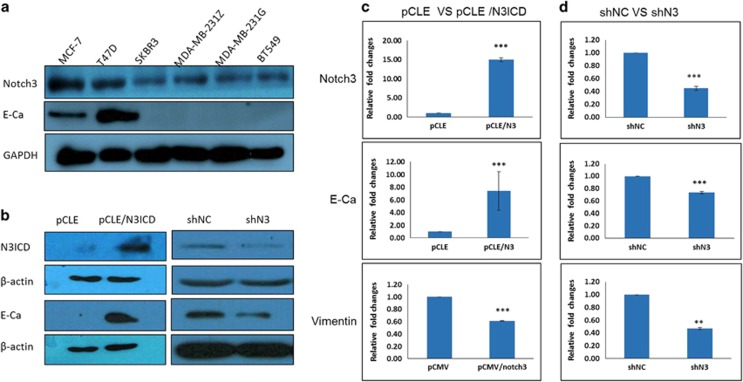
Notch3 expression in various breast cancer cell lines and the correlation between Notch3 and EMT. (**a**) Notch3 expression levels in various human breast cell lines, including MCF-7, T47D, SKBR3, MDA-MB-231 and BT549, were tested via western blotting. (**b**) Confirmation that Notch3 positively regulates E-cadherin expression via western blotting by overexpressing or knocking down Notch3 in MDA-MB-231 cells. (**c**) Confirmation that ectopically overexpressed Notch3 inhibits EMT by qRT-PCR. (**d**) Confirmation that knock down of Notch3 promotes EMT by qRT-PCR. Data are presented as the mean±s.d. of three experiments. *P<0.05, **P<0.01 and ***P<0.001 (Student's *t*-test) as compared with control cells.

We determined relative Notch3 and E-cadherin expression levels via western blotting in five human breast cancer cell lines including MCF-7, T47D, SKBR3, MDA-MB-231(two strains stored in different laboratory) and BT549, of which MCF-7 and T47D are characterized as ER-/PgR-positive luminal mammary carcinoma, MDA-MB-231 and BT549 are characterized as triple-negative/basal-B mammary carcinoma (TNBC), and SKBR3 is a human breast cancer cell line that overexpresses the Her2 (Neu/ErbB-2) gene product. Western blotting revealed that the expression levels of Notch3 and E-cadherin varied among the five breast cancer cell lines. In detail, those tumor cell lines with high relative malignancy, such as TNBC, MDA-MB-231 and BT549, presented lower expression levels of Notch3 and E-cadherin; conversely, the ER-positive hormone-dependent breast cancer cell lines MCF-7 and T47D presented higher expression levels of Notch3 and E-cadherin. These results reveal that cell lines with higher expression levels of Notch3 and E-cadherin tend to have lower relative malignant behavior and vice versa (Figure 1a). Furthermore, these data also indicate that Notch3 expression may be positively associated with that of E-cadherin in breast cancer. A previous study showed that loss of E-cadherin expression is one of the earliest steps in EMT, and is a hallmark of EMT.^25^ To elucidate the effects of Notch3 on EMT progression in breast cancer cells, we first established a N3ICD stable transfectant in low Notch3-expressing MDA-MB-231 cells by co-transfecting pCLE/N3ICD and pEGFP-N plasmids. Meanwhile, we also generated stable Notch3 knockdown cells in high Notch3-expressing MCF-7 cells by transfecting pGPU6/GFP/Neo/shRNA-N3. Western blotting was performed to verify ectopic Notch3 expression and the efficiency of Notch3 knockdown. The results showed that upregulated Notch3 expression was observed in N3ICD/MDA-MB-231 cells; by contrast, downregulated Notch3 expression was evident in shRNA-N3ICD/MCF-7 cells (Figure 1b). With overexpression or knockdown of Notch3, the expression level of E-cadherin correspondingly increased or decreased at both mRNA and protein levels (Figures 1b–d). Taken together, these data indicate that Notch3 may have an inhibitory role during EMT in breast cancer.

### Endogenous Notch3 was downregulated in TGF-β-treated MCF-7 cells

**Figure 2:**
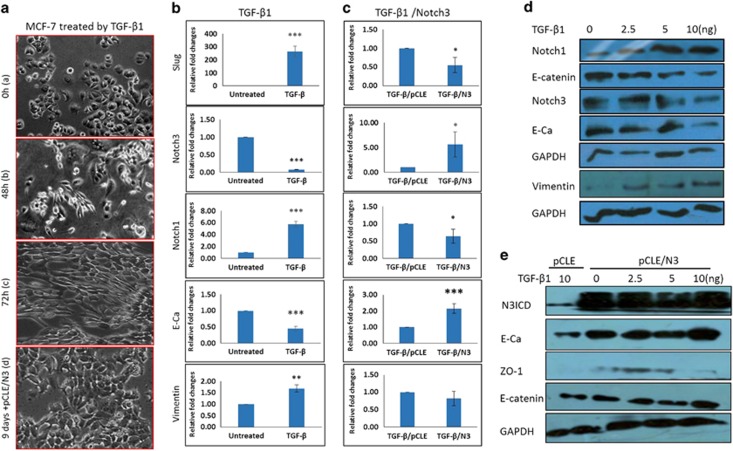
Notch3 can reverse EMT induced by TGF-β1. (**a**) Morphological changes of MCF-7 cells before and after TGF-β1 treatment as well as TGF-β1 treatment combined with N3ICD overexpression. (**b**) RT-PCR confirms that MCF-7 cells undergo EMT after TGF-β1 treatment. (**c**) Western blotting confirms that MCF-7 cells undergo EMT after TGF-β1 treatment. (**d**) RT-PCR confirms that ectopically overexpressed N3ICD can reverse EMT induced by TGF-β1 in MCF-7 cells. (**e**) Western blotting confirms that ectopically overexpressed N3ICD can reverse EMT induced by TGF-β1 in MCF-7 cells. Data are presented as the mean±s.d. of three experiments. *P<0.05, **P<0.01 and ***P<0.001 (Student's *t*-test) as compared with control cells.

To better clarify the relationship between Notch family members and EMT, we established an EMT model in MCF-7 cells that has all the hallmarks of epithelial cells by adding TGF-β1 to cell culture, as TGF-β1 is considered the most potent profibrogenic cytokine. After TGF-β1 treatment for 2 weeks with various concentrations (0.25, 5, 10 and 20 ng), MCF-7 cells underwent a shape change from epithelial-like to spindle-like (mesenchymal morphology; Figures 2a (a–c)). The robust decrease in E-cadherin and E-catenin expression as well as an increase in vimentin expression were found at both the protein and mRNA levels (Figures 2b and d). These results indicate that MCF-7 cells undergo progressive EMT and that our model could be used for further study of the relationship between Notch3 and EMT. Using this model, we found that the expression of Notch3 gradually decreased at both the protein and mRNA levels. By contrast, we noted that TGF-β1 treatment led to obvious upregulation of Notch1 expression (Figures 2b and d). These data indicate that the expression level of Notch3 is indeed inversely related to EMT progression. We therefore speculate that Notch3 may antagonize EMT induced by TGF-β1 in MCF-7 cells.

### Overexpressing Notch3 reverses TGF-β-induced EMT by activating canonical Notch signaling

As our prior experiments established an inverse correlation between Notch3 expression and EMT, we sought to determine whether Notch3 can in fact inhibit EMT in breast cancer epithelium. To directly assess the role of Notch 3 in EMT, we transfected pCLE and pCLE/N3ICD into MCF-7 cells treated with TGF-β1. Interestingly, as shown in Figure 2a (d), the shape of MCF-7 cells treated with TGF-β1 changed from spindle-like back to epithelial-like after N3ICD overexpression. Furthermore, the expression levels of E-catenin, ZO-1 and E-cadherin were significantly upregulated in cells transfected with pCLE/N3ICD as compared with cells transfected with the pCLE (Figures 2c and d). In contrast, the expression levels of Slug and vimentin were sharply downregulated (Figures 2c and d). Taken together, these results suggest that overexpressing Notch3 can reverse EMT induced by TGF-β1.

### Overexpressing N3ICD may inhibit EMT by activating the Salvador–Warts–Hippo pathway in a RBP-Jκ-dependent manner

In our earlier study, *WWC1* (Kibra) was shown to be a differentially expressed gene that was upregulated in MDA-MB-231 cells transfected with pCLE/N3ICD as compared with cells transfected with pCLE by performing transcriptome analysis based on an Affymetrix gene ChIP (Santa Clara, CA, USA; data not shown). Recently, it has been reported that reduced Kibra expression correlates with EMT features in primary breast cancer specimens.^26^ Combined with our prior findings, these results prompted us to examine whether Notch3 inhibition of EMT was mediated by Kibra.

**Figure 3:**
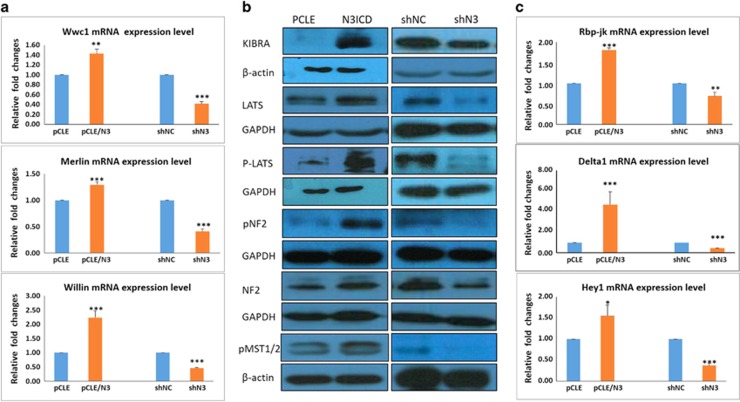
Kibra is regulated by Notch3 in a classical Notch signaling activation pattern. (**a**) RT-PCR confirms that ectopically overexpressed N3ICD can regulate upstream components of the Hippo/YAP signaling pathway. (**b**) Western blotting confirms that ectopically overexpressed N3ICD or Notch3 knockdown can activate or inhibit the core kinase cassette, respectively. (**c**) RT-PCR confirms that ectopically overexpressed N3ICD can activate the canonical signaling pathway. Data are presented as the mean±s.d. of three experiments. *P<0.05, **P<0.01 and ***P<0.001 (Student's *t*-test) as compared with control cells.

The results from our prior transcriptome analysis were further verified by RT-PCR and western blotting. We also detected each component in the SWH (Salvador–Warts–Hippo) signaling pathway. As shown in Figure 3a, the expression levels of the upstream molecules in this pathway, such as Kibra, FRMD6/Willin and Merlin/NF2, were upregulated or downregulated at both the transcriptional and protein levels with overexpressing or knocking down N3ICD in MDA-MB-231 breast cancer cells, respectively. At the same time, western blotting also revealed that the expression levels of core kinase cassette proteins in this pathway, such as pLATS and pMST1/2, were positively correlated with the expression level of Notch3 (Figure 3b); namely, overexpressing N3ICD resulted in a robust increase in Lats1/2 and MST1 kinase phosphorylation in MDA-MB-231/pCLE/N3ICD cells as compared with control cells. Very consistently, we also detected reduced expression of phosphorylated Lats1/2 and MST1 kinase in MCF-7 cells when Notch3 was knocked down, as compared with control cells. These results suggested that overexpressing Notch3 can activate the SWH signaling pathway.

**Figure 4:**
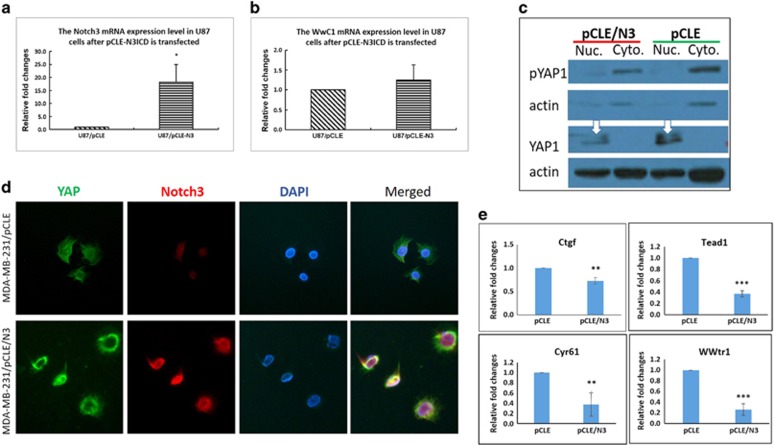
YAP target genes are downregulated when the Hippo/YAP signaling pathway is activated by ectopically overexpressed N3ICD. (**a** and **b**) RT-PCR confirms that ectopically overexpressed N3ICD-induced upregulation of Kibra expression is epithelial cell specific, as this phenomenon does not occur in the U87 glioblastoma cell line. (**c** and **d**) Western blotting and immunofluorescent staining confirm that non-phosphorylated YAP is excluded from the nucleus (the white arrows show non-phosphorylated YAP in the nucleus by western blot) and then is phosphorylated and degraded in MDA-MB-231 cells ectopically overexpressing N3ICD as compared with control cells. (**e**) RT-PCR confirms that YAP target genes are downregulated once the Hippo/YAP signaling pathway is activated by ectopically overexpressing N3ICD. Data are presented as the mean±s.d. of three experiments. *P<0.05, **P<0.01 and ***P<0.001 (Student's *t* test) as compared with control cells.

It remained to be determined whether Notch3 regulates SWH signaling pathway via the canonical Notch signaling pathway; we therefore tested whether a series of molecules in the classical Notch signaling pathway were activated. The expression levels of Notch receptor family members (Notch1–4), their five ligands (Jag1, 2 and DLL1, 3, 4), and six canonical Notch target genes of the Hes/Hey family (*Hes1*, *Hes5*, *Hes7*, *Hey1*, *Hey2* and *HeyL*) were detected by real-time RT-PCR. Of the five possible Notch ligands, only DLL1 was detected at significant levels with overexpressing or knocking down Notch3. To understand which downstream molecules in the Notch signaling pathway are important after Notch 3 overexpression, the expression levels of RBP-Jκ and classical RBP-Jκ-dependent Notch target genes were examined. Of the six possible targets within the Hes/Hey family, only Hey1 was identified (Figure 3c). These data indicate that after overexpressing the N3ICD-activated classical Notch signaling pathway, N3ICD preferentially regulates *Hey1* by activating Delta1, but not *Hes1*. Moreover, to address whether Notch3 regulation of Kibra is specific to epithelial cells, we also tested the expression level of Kibra in the glioma U87 cell line after overexpressing N3ICD. However, the expression level of Kibra did not change with overexpressing N3ICD (Figures 4a and b). Taken together, these data suggest that overexpressing Notch3 inhibits EMT by activating upstream molecules in the SWH pathway in a RBP-Jκ-dependent manner only in breast epithelial cells.

### Overexpressing N3ICD affects YAP phosphorylation and subcellular localization as well as downstream target gene expression

Several studies have shown that TEAD transcription factors and their transcriptional co-activators YAP and TAZ promote EMT,^27^ and loss of Kibra expression leads to EMT features that are concomitant to decreased LATS and YAP phosphorylation.^26^ Considering that only dephosphorylated YAP/TAZ localize in the nucleus and function as transcriptional coactivators for the TEAD family of transcription factors to induce gene expression, we tested and compared the expression levels of dephosphorylated YAP1 in the nucleus between MDA-MB-231/pCLE and MDA-MB-231/pCLE/N3ICD cells by western blotting as well as the expression levels of downstream target genes by RT-PCR.

Western blot analysis of nuclear and cytoplasmic fractions showed that the expression level of YAP1 in the nucleus of MDA-MB-231 cells transfected with pCLE/N3ICD was significantly lower than that in MDA-MB-231 cells transfected with pCLE (Figure 4c; white arrows). These results suggest that the majority of YAP1 is translocated from the nucleus to the cytoplasm.

We also analyzed the YAP1 expression pattern by immunofluorescence staining in MDA-MB-231 cells overexpressing N3ICD and control cells. Double labeling with N3ICD and YAP1 showed that cells in the control group had very weak Notch3 positivity in the nucleus (red color) and had homogeneous nuclear and cytoplasmic YAP1 (green color). By contrast, MDA-MB-231 cells overexpressing N3ICD had very strong Notch3 positivity in the nucleus, whereas YAP1 was excluded from the nucleus such that nuclear YAP1 was very low to undetectable. Most YAP1 positivity was aggregated on the nuclear membrane (Figure 4d). These results suggest that overexpressing N3ICD leads to phosphorylation of the majority of YAP1 by the core kinase cassette, such as pLATS and pMST1/2, and Hippo/YAP pathway activation. In addition, it has been reported that several genes are regulated by YAP1, including connective tissue growth factor, Cyr61, WWTR1 and TEAD. We next examined the expression levels of these genes by RT-PCR. As shown in Figure 4e, overexpressing N3ICD was sufficient to suppress the expression of these genes at the mRNA level. Together, these results suggest that N3ICD is a positive regulator of the Hippo/YAP1 signaling pathway.

### Notch3 regulates Kibra expression at the transcriptional level

**Figure 5:**
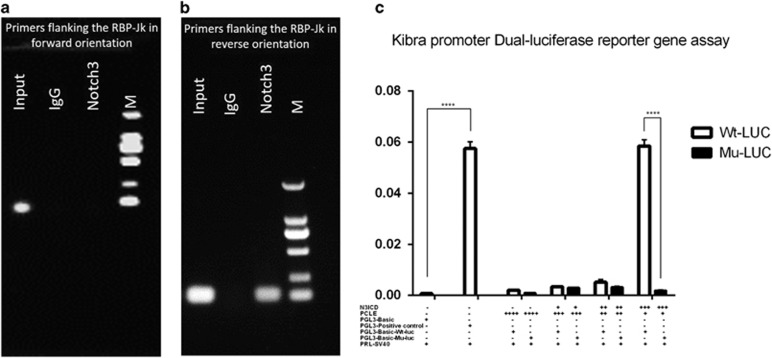
ChIP and luciferase assays confirm that Kibra is regulated by Notch3 at the transcriptional level. (**a**) The ChIP assay used normal IgG (IgG) or anti-Notch3 antibody to determine whether Notch3 can bind the RBP-Jk binding site in the Kibra promoter in MDA-MB-231 cells. After ChIP, PCR shows that the Notch3/RBP-Jk does not bind to the RBP-Jk binding site in the sense orientation. (**b**) After ChIP, PCR reveals that the Notch3/RBP-Jk complex binds to the RBP-Jk binding site in the antisense orientation. (**c**) MDA-MB-231 cells were co-transfected with pCLE, pCLE/N3, pGL-Wt-Luc or pGL-Mut-Luc. All cells were also co-transfected with a Renilla luciferase plasmid. Luciferase activity was normalized to that of Renilla. *P<0.05, **P<0.01, ***P<0.001 and ****P<0.0001 (Student's *t*-test) as compared with control cells. Data are presented as mean±s.e.m. (*n*=3).

Because mammalian Notch proteins enter the nucleus and function as transcription factors by interacting with a single DNA-binding protein (CSL), thereby binding to the RBP-Jκ-binding site contained in the promoter region of a target gene, we next analyzed the promoter sequence of Kibra. With the exception of a canonical RBP-Jκ-binding sites at a position between −1621 to −1614 bp on the sense strand, a putative RBP-Jκ-binding site between −1234 to −1227 bp relative to the transcriptional start site was found on the antisense strand of the DNA. This analysis indicates that Notch3 might act as an upstream inducer of Kibra, most likely in a direct manner. To investigate whether Notch3/RBP-Jκ could form a complex with the Kibra promoter and to define which binding site is eligible for the N3ICD/RBP-Jκ transcriptional activator complex, we performed a chromatin immunoprecipitation (ChIP) assay with primers covering the RBP-Jκ-binding sites in the Kibra promoter region in a Notch3-overexpressing MDA-MB-231 cell line. The Notch3 antibody was used to identify the Notch3/RBP-Jκ-binding site on the Kibra promoter; nonspecific IgG (IgG) was used as a negative control and input was used as a positive control. First, we analyzed the proximal RBP-Jκ-binding site on the sense strand of DNA located −1614 bp upstream of the transcriptional start site of the Kibra gene using primer set 1. As compared with nonspecific IgG-treated cells, the Notch3/RBP-Jκ complex did not bind to this RBP-Jκ-binding site (data not shown). To test whether the Notch3/RBP-Jκ complex binds to the RBP-Jκ-binding site on the antisense strand of DNA located at −1227 bp, we performed a ChIP assay using primer set 2. As expected, binding was observed (Figure 5a). This suggests that Notch3 activates Kibra expression by binding to the RBP-Jκ-binding site on the antisense strand of the Kibra promoter.

On the basis of our ChIP results, we constructed a luciferase reporter vector that included the region from −1296 to −1199 of the Kibra gene (pGL3/Kibra-promotor reporter plasmid). We also generated a mutant luciferase reporter vector of the Kibra promoter by site-directed mutagenesis in which the RBP-Jκ-binding site on the antisense strand of DNA was deleted. A dual-luciferase reporter assay was used to investigate Kibra promoter activity in Notch3-low expressing MDA-MB-231 cells by transient co-transfection with the plasmid-containing Kibra promoter, as well as a Renilla luciferase reporter vector with or without co-expression of the N3ICD-expression vector. Kibra promoter activity increased 2.12- to 35.48-fold in the MDA-MB-231 cell line in a dose-dependent manner, suggesting that the RBP-Jκ-binding site on the antisense strand of DNA is sufficient to elicit N3ICD-dependent gene expression of Kibra. By contrast, the mutant Kibra promoter significantly affected Kibra promoter activity (Figure 5b; *P*<0.001). These results demonstrate that Notch3 directly activates Kibra transcription by specifically binding to the promoter region of the *Kibra* gene.

### The effect of Notch3 on inhibiting EMT can be countered by knocking down Kibra expression

**Figure 6:**
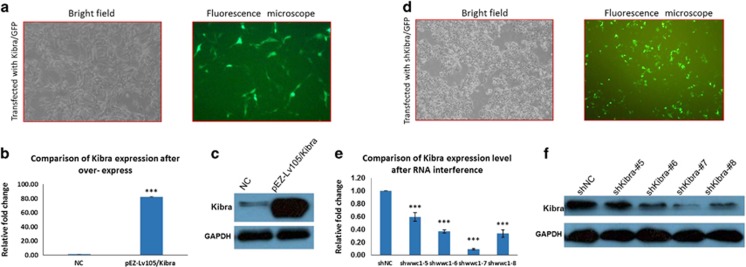
Confirming the ectopic overexpression and knockdown efficiency of Kibra. (**a**) The Kibra overexpression construct was engineered, and its successful transfection is shown by GFP expression. (**b**) RT-PCR confirms that the expression of Kibra mRNA is upregulated after MDA-MB-231 cells are transfected with pCMV plasmids. (**c**) Western blotting confirms that the expression of Kibra protein is upregulated after MDA-MB-231 cells are transfected with pCMV plasmids. (**d**) The Kibra knockdown construct was engineered and its successful transfection is shown by GFP expression. (**e**) RT-PCR confirms that the expression of Kibra mRNA is downregulated after MDA-MB-231 cells are transfected with each of the four knockdown plasmids, shKibra #5–#8. (**f**) Western blotting shows that Kibra protein in MDA-MB-231 cells is reduced after each of the four knockdown plasmids is transfected. Data are presented as the mean±s.d. of three experiments. *P<0.05, **P<0.01 and ***P<0.001 (Student's *t*-test) as compared with controls.

To further understand the role of Kibra in Notch3-induced EMT inhibition in breast cancer cells, we constructed a Kibra expression vector, pEZ-Lv105/Kibra. Plasmids were transiently transfected into low Kibra-expressing MDA-MB-231 cells. GFP-positive cells were used to confirm successful transfection (Figure 6a). RT-PCR and western blotting showed that the expression level of Kibra in MDA-MB-231 cells transfected with pEZ-Lv105/Kibra increased at both the mRNA and protein levels as compared with cells transfected with pEZ-Lv105 (Figures 6b and c). In addition, we also generated four stable Kibra knockdown vectors (pGPU6/GFP/Neo/shRNA-Kibra #5, #6, #7 and #8) and scrambled control (pGPU6/GFP/Neo/shRNA-NC), and transfected them into MCF-7 cells. As shown in Figure 6d, GFP-positive cells were used to observe successful transfection. Accordingly, RT-PCR and western blotting revealed that the expression level of Kibra in shRNA-Kibra/MCF-7 cells was downregulated as compared with shRNA-NC/MCF-7 cells (Figures 6e and f).

**Figure 7:**
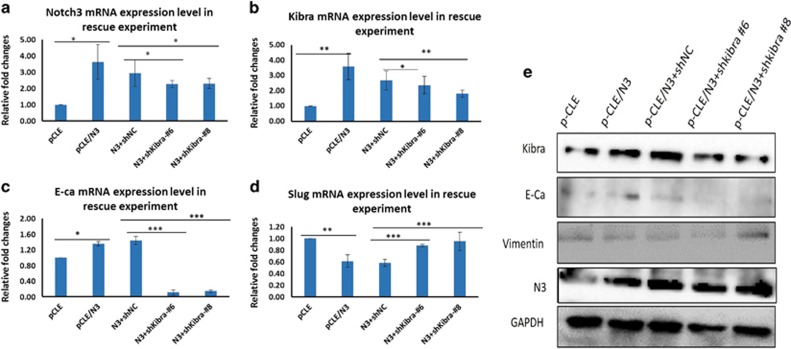
The effect of Notch3 inhibiting EMT can be counteracted by knocking down Kibra with shRNA. (**a**–**d**) After MDA-MB-231 cells were first transfected with pCLE/N3 for 48 h, Kibra expression was knocked down through a second transfection with shKibra #5–#8. RT-PCR results reveal that the effect of Notch3 inhibiting EMT was significantly attenuated at the transcriptional level as demonstrated by the expression of Notch3, Kibra, E-cadherin and slug mRNA. (**e**) MDA-MB-231 cells were subjected to the above-mentioned co-transfection. Kibra expression was knocked down through transfection with shKibra #5–#8. The effect of Notch3 inhibiting EMT was significantly attenuated at the protein level as demonstrated via western blotting to test the expression of Notch3, Kibra, E-cadherin and vimentin. *P<0.05, **P<0.01 and ***P<0.001 (Student's *t*-test) as compared with control cells. Data are presented as mean±s.e.m. (*n*=3)

Next, to further validate that Notch3 inhibition of EMT through the Hippo/YAP pathway is mediated by Kibra, we performed rescue experiments by co-transfecting MDA-MB-231 cells with plasmids encoding N3ICD and shKibra #6 or #8; we hypothesized that this would reduce the effects of N3ICD. Notably, as shown in Figures 7a–d, 48 h after transfection, RT-PCR analysis indicated that the upregulated expression of Kibra and E-cadherin induced by N3ICD was significantly attenuated by shKibra #6 or #8 in N3ICD-expressing MDA-MB-231 cells as compared with cells cotransfected with shRNA-NC. Conversely, N3ICD-inhibited vimentin and slug expression was reproducibly increased after shKibra#6 or #8 treatment. Furthermore, N3ICD-induced EMT inhibition was attenuated when the shKibra #6 or #8 was introduced. These results were further verified by western blot analysis (Figure 7e). Collectively, our data demonstrate that Kibra is required for N3ICD-induced EMT inhibition. Unexpectedly, we found that the expression level of Notch3 was sharply reduced when shKibra#6 or #8 was introduced.

### Functional relationship between Notch3 and Kibra in breast cancer epithelial cells

**Figure 8:**
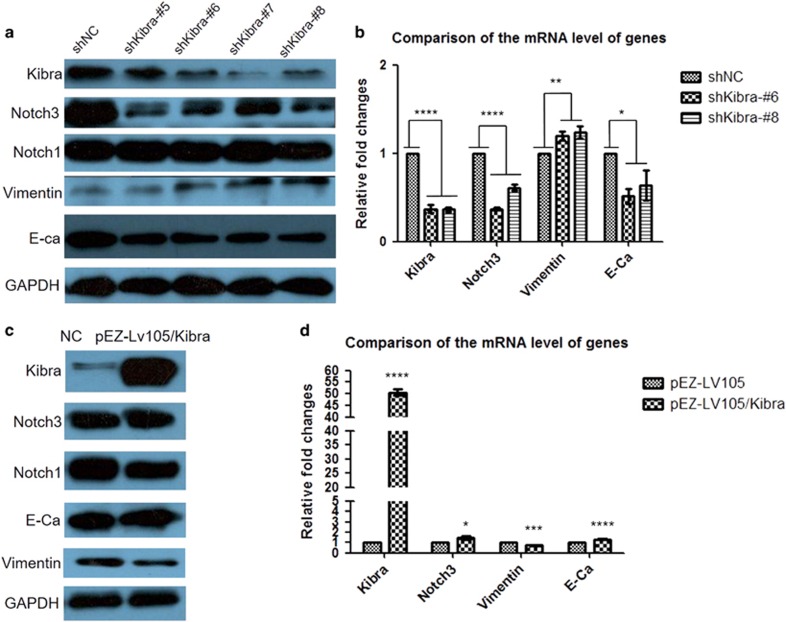
Functional relationship between Notch3 and Kibra in breast cancer epithelial cells. (**a**) After shKibra constructs are transfected into MCF-7 cells, the expression level of Notch3 is significantly reduced. Correspondingly, significantly elevated vimentin and decreased E-cadherin expression levels are seen as compared with those of MCF-7 cells transfected with shNC. However, the expression of Notch1 is unchanged. (**b**) RT-PCR shows that the expression levels of Notch3 and E-cadherin mRNA are downregulated in MDA-MB-231 cells after shKibra#6 and #8 are transfected, whereas the expression level of vimentin was upregulated. (**c**) Nevertheless, when Kibra overexpression constructs are transfected into MDA-MB-231 cells, the expression of Notch3 is slightly upregulated (or unchanged), and the expression levels of vimentin and E-cadherin have no significant changes. The expression of Notch1 is only slightly inhibited. (**d**) RT-PCR analysis reveals that the expression levels of Notch3 and E-cadherin mRNA are upregulated in MDA-MB-231 cells, whereas the expression of vimentin mRNA is reduced when Kibra overexpression constructs are transfected into MDA-MB-231 cells. Data are presented as the mean±s.d. of three experiments. *P<0.05, **P<0.01, ***P<0.001 and ****P<0.0001 (Student's *t*-test) as compared with control cells.

To investigate whether there is any interaction between Notch3 and Kibra in breast cancer epithelial cells, we first analyzed the expression of Notch3 when overexpressing or knocking down Kibra. shKibra constructs were transfected into MCF-7 cells. The expression level of Notch3 was determined by western blotting. As shown in Figure 8a, the expression level of Notch3 was indeed significantly reduced when Kibra was knocked down. Correspondingly, significantly elevated vimentin and decreased E-cadherin expression levels were observed in MCF-7/shKibra (all four target sites) cells as compared with MCF-7/shNC cells. We also noted that Notch1 expression was unchanged. These results were confirmed by RT-PCR (Figure 8b). Nevertheless, when Kibra overexpression constructs were transfected into MDA-MB-231 cells, western blot data revealed that the expression of Notch3 was slightly upregulated (or unchanged); the expression of vimentin and E-cadherin was similarly unchanged, and the expression of Notch1 was slightly inhibited (Figure 8c). These results were supported by RT-PCR analysis (Figure 8d). Collectively, our findings demonstrate that Kibra is necessary for maintaining normal expression of Notch3 in breast cancer epithelial cells, but an additional signal may be required to fully regulate Notch3 expression.

## Discussion

Breast cancer is the most common cancer in women worldwide. Despite therapeutic advances made in recent decades, about 20% of patients will experience metastasis, recurrence, chemoresistance or even death; TNBC accounts for a much higher proportion of breast cancer-related mortality than other types of breast cancer.^28^ Thus, there is an urgent need to find effective targeted agents for the treatment of this disease.^29^ Although no single genetic or metabolic state could be considered critical for the formation and progression of TNBC, we assert that there must be some common molecular characteristics of TNBC. Hence, it is imperative to understand the molecular characteristics of TNBC in order to develop more effective treatments for this cancer.

Interestingly, in this study, one of the most remarkable findings is the heterogeneity of Notch3 expression in various breast cancer cell lines. Tumor cell lines with high proliferation rates, poor differentiation and an aggressive clinical course, such as the TNBC cell lines MDA-MB-231 and BT549, presented lower expression levels of Notch3 and E-cadherin. In contrast, some ERα^+^ breast cancer cell lines with low proliferation rates, such as MCF-7 and T47D, presented higher expression levels of Notch3 and E-cadherin. At present, it is generally accepted that EMT-inducing factors initiate epithelial re-organization by impairing the expression of E-cadherin.^30^ Our observations suggest that the expression levels of Notch3 and E-cadherin are positively correlated in breast cancer epithelial cells. We therefore hypothesized that, in total contrast to Notch1, Notch3 may act to inhibit EMT in breast cancer epithelial cells.

This idea is further supported by several of our observations. First, we established an EMT model by treating MCF-7 cells with TGF-β1 because TGF-β1 is a potent inducer of EMT both during development and in cancer.^31, 32, 33^ In this model, the expression levels of Notch3 and E-cadherin exhibited TGF-β1 dose-dependent decreases, and the expression of vimentin exhibited a TGF-β1 dose-dependent increase. Second, by contrast, the expression of Notch1 was gradually upregulated with EMT initiation, consistent with a previous report that the activation of Notch1 signaling contributes to the acquisition of the EMT phenotype.^34^ Third, enforced N3ICD expression in TGF-β1-treated MCF-7 cells promoted a morphological reverse from mesenchymal-to-epithelial-like, upregulated the expression of E-cadherin and inhibited the expression of vimentin. Nevertheless, we did not observe that Notch1 had similar effects. These data confirmed our hypothesis that Notch3 is a potent inhibitor of EMT in breast cancer epithelial cells. We therefore conclude that, in TNBC, the loss of Notch3 expression may be one of the most important genetic traits of this tumor subtype. Hence, it is imperative to understand the molecular mechanism of Notch3 function to treat TNBC.

To thoroughly unravel the molecular events that Notch3 signaling may induce to inhibit EMT, newly available transcriptomic tools allowed us to explore and compare the variation in gene expression between cells ectopically expressing N3ICD (MDA-MB-231/pCLE/N3) and control cells on a genomic scale. An unanticipated discovery of our study is that upregulation of the WW domain protein Kibra was one of the prominent features of MDA-MB-231/pCLE/N3ICD cells as compared with control cells.

Genetic screens have identified that Kibra acts upstream of Hippo in *Drosophila melanogaster*.^35^ It functions together with the tumor suppressors Merlin (Mer; also known as NF2 for neurofibromatosis-2) and Expanded (Ex) to activate Lats1/2 (Wts orthologs) in a cooperative manner and regulate Hippo signaling activity in *Drosophila*.^36, 37, 38^ The core kinase cassette of the mammalian Hippo pathway includes STE20 family protein (MST) kinases (MST1 and MST2) and large tumor suppressor (LATS) kinases (LATS1 and LATS2).^39^ When the pathway is activated, MST kinases phosphorylate LATS kinases, which then phosphorylate the transcriptional co-activators YAP and/or TAZ,^40^ thereby inactivating YAP.^41^ This phosphorylation of YAP at serine 127 (S127) prevents its nuclear shuttling and inhibits expression of YAP target genes.^42^ By contrast, non-phosphorylated and therefore active YAP enters the nucleus and binds to transcription factors.^43^ Recently, the Hippo pathway has been shown to control organ size, promote cell death and differentiation, and inhibit cell proliferation; therefore, the Hippo pathway may function as a key node to coordinate these cellular processes.^44, 45^ More recently, activated YAP/TAZ has been shown to promote EMT and cell migration.^46^ Thus, Hippo/YAP pathway activation mediated by Kibra upregulation could have a key role in reversing EMT in MDA-MB-231/pCLE/N3ICD cells. Therefore, we hypothesized that ectopic N3ICD overexpression may reverse EMT by activating Hippo/YAP pathway-mediated Kibra upregulation in breast cancer epithelial cells.

Kibra is mainly localized to the apical membrane domain of epithelial cells and is a component of adherens junctions (which include Kibra, NF2, α-catenin and E-cadherin).^47^ However, the regulation of Kibra, particularly by Notch3, has not been addressed in prior studies. Notch proteins are evolutionarily conserved transmembrane receptors that are activated by transmembrane ligands expressed at the surface of adjacent cells.^48^ Aside from their function as transcription factors, short-range intercellular communication mediated by Notch proteins has a crucial role in embryonic development and tissue renewal. For instance, Notch can promote the expression of the tight junction molecule Crb in *Drosophila* wing epithelial discs.^49^ Furthermore, it is well known that the classical Notch signaling pathway is regulated in a stepwise processes, and that the NICD (intracellular domain of Notch) is released from the membrane and enters the nucleus to form a transcriptional complex with the RBP-Jκ transcription factor (RBP-J, Su(H), Lag-1). RBP-Jκ is a 60 kDa DNA-binding protein that recognizes a consensus sequence, although it has no typical DNA-binding motif. In the absence of a Notch signal, it is thought that RBP-Jκ functions as a repressor by interacting with transcriptional co-repressor proteins. When NICD is overexpressed, it binds to RBP-Jκ, displaces the repressive cofactors bound to RBP-Jκ and recruits a transcriptional activator complex, which initiates transcription of downstream target genes.^48^ The canonical recognition sequence for RBP-Jκ was originally defined as 5′-GTGGGAA-3′, but functional variants of this sequence have since been described, and it is likely that sequence context is important.^50, 51, 52^ In this study, we found two putative RBP-Jκ-binding sites in the promoter of Kibra, of which one is the on the sense strand (−1621 to –1614 bp) and the other is on the antisense strand (5′-TTCCCAC-3′ −1234 to –1227 bp). Hence, it is reasonable to hypothesize that Kibra is regulated by Notch3.

This hypothesis further supported by a series of experiments. (1) The expression levels of RBP-Jκ, Delta1 and Kibra increased and decreased with Notch3 gain- and loss-of-function, respectively, using gene transfection and RNAi. These data suggest that Kibra regulation by N3ICD is classical Notch signaling pathway-dependent in MDA-MB-231/pCLE/N3 cells. (2) The Kibra promoter sequence includes an antisense RBP-Jκ-binding site, and that Kibra was transcriptionally regulated by N3ICD was confirmed using ChIP and a dual luciferase reporter gene assays. (3) A series of upstream components of the Hippo/YAP pathway, including Merlin, Willin and the core kinases, such as LATS1/2, MST1/2, were upregulated or downregulated with overexpressing or knocking down Notch3, respectively. (4) Regarding subcellular localization of YAP, both immunofluorescence staining and western blots on both cytosolic and nuclear fractions showed that active YAP was excluded from the nucleus upon N3ICD overexpression. (5) YAP target molecules such as TEAD1, connective tissue growth factor, TAZ and CYR61 were significantly downregulated at the transcriptional level when N3ICD was ectopically overexpressed. (6) EMT inhibition induced by overexpressing N3ICD can be attenuated by knocking down Kibra expression in MDA-MB-231/pCLE/N3 cells. Collectively, this study identifies that N3ICD overexpression is sufficient to upregulate Kibra and thereby inhibit EMT through Hippo/YAP pathway activation.

Quite intriguingly, in our ChIP experiment, real-time PCR analysis using specific primers flanking the RBP-Jκ-binding site in the antisense orientation showed a very distinct PCR product. Nevertheless, we did not get any positive product using primers flanking the classical RBP-Jκ-binding site in the forward orientation under the same conditions. Our observations strongly suggest that N3ICD/RBP-Jκ mainly binds to the RBP-Jκ-binding site in the antisense orientation. Accordingly, the ability of RBP-Jκ to bind to the RBP-Jκ-binding site in the antisense orientation had been demonstrated in a previous study.^53^ Furthermore, studies have shown that all four Notch proteins share substantially similar domain architectures, and that each Notch protein may target a discrete set of downstream genes.^53^ Possible explanations for this are as follows: (1) the ability of each of the four Notch proteins to activate a given promoter is only partially dependent on cell type; (2) NICD/RBP-Jκ bound to recognition sequences is context, distance and orientation dependent; (3) N3ICD/RBP-Jκ might preferentially bind RBP-Jκ-binding sites in the antisense orientation; and (4) the cooperative interaction between RBP-Jκ molecules in which two RBP-Jκ molecules are properly positioned in proximity to each other can lead to a higher probability of Notch activation. We are fascinated by this question and, accordingly, these possibilities certainly merit further study.

It should be noted that, contrary to the apparent direct connection between Notch1/2 signaling and the Hippo/YAP pathway in several systems, such as the developing embryo,^54^ neuronal stem cells,^55^ skeletal muscle,^27^ hepatocyte-derived stem cells and mature hepatocytes,^56, 57^ there has been little available evidence on the correlation between the Notch3 and Hippo/YAP pathways, particularly in breast epithelial cells. It is quite clear that YAP1 is a direct target of N1ICD, and ectopically overexpressed Notch1 can inactivate Hippo-YAP signaling and promote EMT.^55^ Nevertheless, based on our results, Notch3 has an opposite effect on the Hippo/YAP pathway.

Not surprisingly, there is growing evidence that different Notch molecules may hold different functions even though they are all in the same family. For example, a recent study demonstrates that activated Notch enhances tumor survival by maintaining the cancer stem cell pool, positively regulating EMT by upregulating slug and snail expression, and contributing to chemo/radioresistance.^58, 59^ In breast cancer, elevated levels of Jag1 and Notch1 correlate with poor prognosis and survival,^60^ and activated Notch4 can transform mouse mammary epithelium.^61, 62^ In addition, it has recently been shown that Notch1 and Notch3 mediate cellular senescence, revealing a novel function of Notch in tumor suppression.^63, 64^ It is noteworthy that Lin *et al.*^65^ characterized two regions within the Notch3 extracellular domain containing EGF receptor-like repeats that may be responsible for the distinct effects of Notch3 versus those of Notch1 in non-small-cell lung cancer. Consequently, further study is necessary to fully elucidate the role of Notch3 signaling in breast cancer.

It is somewhat surprising that the expression of Notch3 can be regulated by Kibra, particularly in the case of knocking down Kibra with shRNA where the expression of Notch3 was sharply reduced. However, ectopically expressing Kibra had no significant effect on Notch3 expression levels. Importantly, the expression of Notch1 was not affected. These data suggest the existence of a feedback loop where expression of Notch3 is initially independent of Kibra, but requires Kibra for maintained expression in breast cancer epithelial cells. Therefore, we conclude that Notch3 and Kibra may be key nodes that mediate direct crosstalk between the Hippo/YAP and Notch3 signaling pathways. Moreover, in breast cancer epithelial cells, Kibra might be a point of convergence in the Hippo/YAP signaling pathway as activated by Notch3 to have a pivotal role in inhibiting EMT. Nevertheless, the molecular mechanism by which Kibra modulates Notch3 deserves to be addressed in future studies.

Of note, Kibra, Ex and Mer function together to activate Hippo/YAP signaling. In this study, we noted that Notch3 can upregulate Ex or Mer. One possibility is that these proteins function redundantly and Hippo/YAP pathway receives signals from the same upstream regulator complex. Therefore, our findings do not exclude the possibility that Ex or Mer activate the Hippo/YAP signaling pathway, especially given the recent report that Ex can directly bind YAP.^66^

## Conclusions

**Figure 9:**
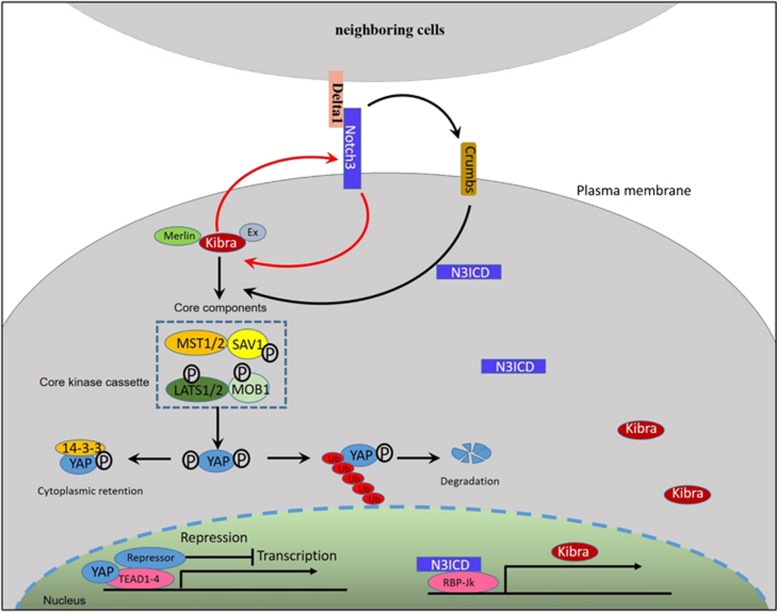
A working model of Notch3 and Kibra co-regulation in breast cancer cells. There is a complex inter-related and inter-dependent correlation between Kibra and Notch3. Loss of Notch3 leads to the decreased Kibra expression at both the transcriptional and protein levels. Downregulated Kibra, through the canonical Hippo kinase cascade, inhibits YAP phosphorylation, contributes to the nuclear accumulation of YAP, initiates target gene transcription and promotes EMT. Furthermore, loss of Kibra leads to downregulation of Notch3 expression. Conversely, ectopically overexpressed Notch3 upregulates Kibra expression, followed by an activated core kinase cassette, inhibited target gene transcription and finally suppressed EMT in breast cancer epithelial cells. In addition, ectopically overexpressed Notch3 upregulates the expression of the apical transmembrane protein Crb (Crumbs), which also interacts with Ex and modulates its localization and stability.

Our findings unequivocally demonstrate that Notch3 can act as a tumor suppressor in breast cancer epithelial cells, and that loss of Notch3 is an important feature of TNBC. We also shed light on the underlying molecular mechanism by which ectopic overexpression of Notch3 can reverse EMT in breast cancer epithelial cells. A working model (Figure 9) is proposed for breast cancer cells, particularly TNBC cells, in which there exists complex inter-related and inter-dependent correlation between Kibra and Notch3. Loss of Notch3 leads to reduced expression of Kibra at the transcriptional and protein levels. Downregulated Kibra, through the canonical Hippo kinase cascade, inhibits YAP phosphorylation, contributes to the nuclear accumulation of YAP, initiates target gene transcription, and promotes EMT. Together, these findings deepen our understanding of EMT in both development and disease, and will undoubtedly help to provide new therapeutic strategies for interfering with cancer invasion and metastasis, especially for TNBC. The precise mechanism by which Notch3, Crumbs and Kibra interact and sense external forces, signals from neighboring cells and the extracellular matrix to control EMT is still not fully understood and remains a key question in the field.

## Materials and methods

### Cell lines, antibodies and reagents

MDA-MB-231 and MCF-7 breast cancer cell lines were purchased from the Committee on Type Culture Collection of the Chinese Academy of Science (Shanghai, China). Cells were routinely grown with DMEM supplemented with 10% fetal bovine serum and 1% penicillin/streptomycin. Cells were replicated every 4–5 days and the medium was changed once in the interim. Once these cultures attained confluence, cells were used for various experiments such as RT-PCR and western blotting. The antibodies used in this study are shown in Table 1.

Human recombinant TGF-β1 used in this study was obtained from PeproTech EC Ltd. (Cat# 100-21; London, UK). The working concentration was 10 ng/ml.

### MCF-7 EMT cell model

MCF-7 cells were trypsinized and placed into six-well plates at appreciate cell densities. One day later, cells were treated in 1–2.5% fetal bovine serum-containing medium with or without 10 ng/ml of TGF-β1 (PeproTech EC Ltd.) for 4–6 days.

### Vectors, transient and stable transfection

The eukaryotic expression plasmids pGLE and pGLE-N3ICD were preserved by our laboratory. Notch3-interfering plasmid was made using pGPU6/GFP/Neo (Shanghai GenePharma Co., Ltd, Shanghai, China) as backbone. The shRNA sequences targeting Notch3 mRNA are shown in Table 2. After annealing, the shRNA was inserted into the pGPU6/GFP/Neo vector. pEZ-Lv105, pEZ-Lv105/Kibra, pGPU6/GFP/Neo/shNC and pGPU6/GFP/Neo/shKibra #5, #6, #7 and #8 were purchased from GeneCopoeia (Rockville, MD, USA).

Cells were plated in a six-well plate at a density of 1 × 10^5^ cells per well in 2 ml of the appropriate growth medium supplemented with serum. For each transfection, 1 to 2 μg of DNA was diluted into 100 μl of serum-free medium, and 2 to 25 μl of lipofectamine reagent was diluted into 100 μl of serum-free medium. The two solutions were combined, gently mixed and incubated at room temperature (25 °C) for 15 to 45 min. The cells were washed once with 2 ml of serum-free medium. For each transfection, 0.8 ml of serum-free medium without antibacterial agents was added to each tube containing lipid:DNA complexes. The diluted lipid:DNA solution was mixed gently and overlaid onto washed cells. Growth medium was replaced 6 to 8 h after the start of transfection. For transient transfection, depending on cell type and promoter activity, cell extracts were tested for gene activity 24–72 h after the start of transfection. For stable transfection, after transfection, cells were allowed to grow and express the protein for G418 resistance under non-selective conditions for at least 24 h. For the selection of stably expressing cells, cells were cultivated in standard medium with supplements and the appropriate amount of G418. Cells were grown for at least 3 weeks under selection pressure to avoid contamination with non-resistant cells, and then the G418 concentration was reduced after 1–2 weeks.

### Quantitative real-time PCR

In brief, the RNA was isolated with Trizol reagent according to the manufacturer's instructions. Contaminating DNA was removed using a TURBO DNA-free Kit (Ambion, Austin, TX, USA) and cDNAs were synthesized from 2 μg of total RNA using PrimeScript RT reagent Kit (Takara Bio, Tokyo, Japan) in a 20 μl reaction mixture following the manufacturer's instructions. Real-time PCR was carried out in the Bio-Rad 5-Color System (Hercules, CA, USA). Primers were designed online with IDT Scitools and the sequences are listed in Table 1. To confirm the specificity of PCR products, melting curves were determined using iCycler iQ software (Hercules, CA, USA), and samples were run on an agarose gel. The expression change of a target gene in RCS-p+ rates relative to the control rate was calculated as fold change=2^−(ΔCT,Tg-ΔCT,control)^. The following PCR scheme was used: 5 min at 94 °C, (30 s at 94 °C, 30 s at 63 °C, 30 s at 72 °C) × 35, 10 min at 72 °C and 4 °C thereafter.

### Immunofluorescence staining

MDA-MB-231 cells were transfected with pCLE and pCLE/N3 plasmid then plated on glass coverslips for immunofluorescent microscopic analysis. In detail, the cells were treated for 5 min at 25 °C with 0.5% Triton X-100 followed by 1% bovine serum albumin in phosphate-buffered saline for 1 h without washing. They were then incubated with Notch3 (red) and YAP1 (green) overnight at 4 °C. The following day, sections were washed and incubated with secondary antibodies for 1 h at 25 °C in the dark. After incubation for 5 min at 25 °C with DAPI (Beyotime, Beijing, China), sections were washed, coverslipped with water soluble mounting liquid (Beyotime) and examined using a fluorescence or confocal microscope. The positive area was measured from confocal images of sections with the image-analysis software Image-Pro Plus 6.0 (Media Cybernetics, Silver Spring, MD, USA).

### Western blot

For the detection of protein expression levels, cells were homogenized in ice-cold RIPA lysis buffer (Beyotime). The homogenates were then centrifuged at 12 000 *g* for 5 min at 4 °C. Protein in the clear supernatants was quantified using a bicinchoninic acid kit (Beyotime), and then samples were reduced and stored at −80 °C until use. Samples (70 μg of protein/lane) were separated by 12% SDS-polyacrylamide gel electrophoresis. Proteins were transferred from the gel onto a PVDF (polyvinylidene fluoride) membrane. After transfer, PVDF membranes were blocked with blocking solution containing Tris-buffered saline, 0.1% Tween 20 and 5% fat-free milk for 1 h at 25 °C. Membranes were then incubated with primary antibodies (Table 2) overnight at 4 °C. Next, the membranes were incubated in appropriate secondary antibody for 1 h at 25 °C while shaking. Finally, PVDF membranes were scanned using the Quantity One Imaging System (Bio-rad) for Notch3, Kibra, LATS1, MTS1/2, NF2 and GAPDH bands. Densitometric ratios were obtained to semi-quantify the relative levels of Notch3, Kibra, LATS1, MTS1/2 and NF2. Each experiment was repeated three times. All values are presented as mean±s.d.

### ChIP assay

ChIP assays were performed as previously described.^67^ In brief, MDA-MB-231 cells at 80–90% confluence growing in 10-cm dishes were treated with 1% formaldehyde for 10 min to cross-link proteins to DNA, and then sonicated four times for 10 s using a sonicator with a microtip in a 1.5- ml tube. The resultant lysate underwent immunoprecipitation with 1 μg polyclonal anti-Notch3 antibody. Normal IgG was used as an immunoprecipitation control, and the supernatant was used as an input control. Immunoprecipitated complexes were collected by adding protein A/G-agarose/salmon sperm DNA beads and incubating samples for 2 h at 4 °C. The beads were then treated with RNase A (50 μg/ml) and proteinase K. DNA was extracted with phenol/chloroform and co-precipitated with glycogen, dissolved in 25 μl TE buffer and subjected to PCR amplification for RBP-Jκ-binding sites in the Kibra promoter using specific primers (Table 2). The acquired DNA was resolved on a 2% agarose gel and stained with Goldview (Transgene, Guangzhou, China).

### Site-directed mutation

The pGL-3-Kibra promoter mutant construct was generated by deleting the CLS-binding site in the wild-type pGL-3-Kibra promoter vector using a site-directed mutagenesis kit (Cat. D0206, Beyotime) according to the provided manufacturer specifications. Primers used are shown in Table 2.

### Luciferase reporter assays

For the assessment of RBP-Jκ-binding sites in the Kibra promoter, the MDA-MB-231 cells (1 × 10^3^) were plated in 96-well plates in triplicate, and cells were transiently transfected with pCLE, pCLE/N3, pGL3/Kibra-promoter reporter, pGL3/ΔKibra-promoter reporter and Renilla luciferase reporter plasmids using Lipofectamine 2000. Cells were harvested 48 h after transfection and assayed by methods adapted from Ausebel *et al.*^68^ Cells were washed once with phosphate-buffered saline and lysed in 100 μl of lysis buffer (100 mM KPO_4_ buffer, pH 7.8; 0.2% Triton; 1 mM dithiothreitol; protease inhibitors) at 25 °C for 10 min. In all, 5 μl of lysate was used to determine β-galactosidase concentration to normalize by transfection efficiency. These assays were performed according to the Tropix Galacton chemiluminescent substrate (Thermo Fisher Scientific, Waltham, MA, USA) instructions. In all, 50 μl of lysate incubated with luciferin assay buffer (30 mM Tricine, pH 7.8; 3 mM ATP; 15 mM MgSO_4_; 10 mM dithiothreitol; 0.2 mM CoA; 1 mM luciferin) was used to determine luciferase activity using a Lumat LB 9507 luminometer (Berthold Technologies, Bad Wildbad, Germany).

### Statistical analysis

Statistical analysis was performed using SPSS 16.0 (SPSS Inc., Chicago, IL, USA). Differences between variables were assessed by a two-tailed Student's *t*-test. Data are presented as mean ±s.d. unless otherwise indicated. Two-sided *P*<0.05 was considered statistically significant. Each experiment was performed at least three times.

## Figures and Tables

**Table 1:** Antibodies used in this study

*Antibody*	*Cat.#*	*Company*	*Con*.	*Species*
Anti-NOTCH3 (EPR16623)	ab178948	Abcam (Danvers, MA, USA)	1:1000	Rabbit
Anti-YAP1 (phospho S127)	ab76252	Abcam	1:10000	Rabbit
Anti-YAP1	ab52708	Abcam	1:1000	Mouse
Anti-Willin	ab171745	Abcam	1:1000	Rabbit
Anti-Kibra	sc-133374	Santa Cruz (Santa Cruz, CA, USA)	1:1000	Rabbit
Anti-LATS1	ab70561	Abcam	1:5000	Rabbit
Phospho-LATS1 (Ser909) antibody	9157S	Cell Signaling Technology (Danvers, MA, USA)	1:1000	Rabbit
pMST1/2	PA5-17674	Thermo (Waltham, MA, USA)	1:1000	Rabbit
NF2/Merlin (phospho S518)	ab2478	Abcam	1:1000	Rabbit
NF2 Merlin	ab30329	Abcam	1:1000	Rabbit
GAPDH	TA-08	ZhongshanJinqiao (Beijing, China)	1:3000	Mouse

**Table 2:** Primers used in this study

*Gene/ChIP/luciferase assay*	*Forward primer*	*Reverse primer*
*Notch3*	5′-CTGCTGTTGGACCACTTTGC-3′	5′-CTTTGAGGCCAGGGAGGAAG-3′
*Notch1*	5′-CGCTGACGGAGTACAAGTG-3′	5′-GTAGGAGCCGACCTCGTTG-3′
*Kibra*	5′-AGCTCCAAGTATGACCCTGAG-3′	5′-AAAGCCACGCTCTTTGAACTG-3′
*Merlin*	5′-TTGCGAGATGAAGTGGAAAGG-3′	5′-CAAGAAGTGAAAGGTGACTGGTT-3′
*Ex*	5′-CCACCTCTTTGGACTCAGTGT-3′	5′-CAAATTGGTCGATACCCTTGCT-3′
*CTGF*	5′- ACCGACTGGAAGACACGTTTG-3′	5′- CCAGGTCAGCTTCGCAAGG-3′
*Tead*	5′-GGCCGGGAATGATTCAAACAG-3′	5′- CAATGGAGCGACCTTGCCA-3′
*Cyr61*	5′-CTCGCCTTAGTCGTCACCC-3′	5′-CGCCGAAGTTGCATTCCAG-3′
*Wwtr1*	5′-GATCCTGCCGGAGTCTTTCTT-3′	5′- CACGTCGTAGGACTGCTGG-3′
*E-cadherin*	5′-AAAGGCCCATTTCCTAAAAACCT-3′	5′-TGCGTTCTCTATCCAGAGGCT-3′
*RBP-Jk*	5′-CGGCCTCCACCTAAACGAC-3′	5′-TCCATCCACTGCCCATAAGAT-3′
*Hes-1*	5′-TCAACACGACACCGGATAAAC-3′	5′-GCCGCGAGCTATCTTTCTTCA-3′
*Delta*	5′-GATTCTCCTGATGACCTCGCA-3′	5′-TCCGTAGTAGTGTTCGTCACA-3′
*Hey-1*	5′-ATCTGCTAAGCTAGAAAAAGCCG-3′	5′-GTGCGCGTCAAAGTAACCT-3′
*Crumb3a*	5′-CAGGTGCCTCTCAAATTCTTGC-3′	5′-ACAGGAACCAATGGTAGTTTCAC-3′
*Vimentin*	5′-GACAATGCGTCTCTGGCACGTCT-3′	5′-TCCGCCTCCTGCAGGTTCTT-3′
*GAPDH*	5′-TGGACTCCACGACGTACTCAG-3′	5′-ACATGTTCCAATATGATTCCA-3′
Luciferase assay: wild-type CSL-binding site	5′-CGAGCTCGACCCAACTGGGCTTCATT-3′	5′-TCCCCCGGGGGACCCATCTCCTCTTACATCTGTG-3′
Luciferase assay: mutated CSL-binding site	5′-GGCAAAAAAGGGTTAAATCTCAGTACACAGATGTAA-3′	5′-TTACATCTGTGTACTGAGATTTAACCCTTTTTTGCC-3′
ChIP primers flanking RBP-Jk-binding site in forward orientation	5′-GACCCAAACAGAGGAAGTGTAG -3′	5′-GGCCCTCCTTCATTCCATAAA-3′
ChIP primers flanking RBP-Jk-binding site in reverse orientation	5′-ACCCAACTGGGCTTCATT-3′	5′-CCCATCTCCTCTTACATCTGTG-3′
sh*Notch3*	5′-CACCGTATAGGTGTTGACGCCATCCACGCATT CAAGAGATGCGTGGATGGCGTCAACACCTATATTTTTTG-3′	5'-GATCCAAAAAATATAGGTGTTGACGCCATCCAC GCATCTCTTGAATGCGTGGATGGCGTCAACACCTATAC-3′
